# Whole Exome Sequencing of Cell-Free DNA for Early Lung Cancer: A Pilot Study to Differentiate Benign From Malignant CT-Detected Pulmonary Lesions

**DOI:** 10.3389/fonc.2019.00317

**Published:** 2019-04-24

**Authors:** Tina D. Tailor, Xiayu Rao, Michael J. Campa, Jing Wang, Simon G. Gregory, Edward F. Patz Jr.

**Affiliations:** ^1^Department of Radiology, Duke University Medical Center, Durham, NC, United States; ^2^Division of Quantitative Sciences, Department of Bioinformatics and Computational Biology, The University of Texas MD Anderson Cancer Center, Houston, TX, United States; ^3^Duke Molecular Physiology Institute, Duke University Medical Center, Durham, NC, United States; ^4^Department of Pharmacology and Cancer Biology, Duke University Medical Center, Durham, NC, United States

**Keywords:** cell free DNA, cancer detection, lung cancer—diagnosis, whole exome sequencing, pulmonary nodule

## Abstract

**Introduction:** Indeterminate pulmonary lesions (IPL) detected by CT pose a significant clinical challenge, frequently necessitating long-term surveillance or biopsy for diagnosis. In this pilot investigation, we performed whole exome sequencing (WES) of plasma cell free (cfDNA) and matched germline DNA in patients with CT-detected pulmonary lesions to determine the feasibility of somatic cfDNA mutations to differentiate benign from malignant pulmonary nodules.

**Methods:** 33 patients with a CT-detected pulmonary lesions were retrospectively enrolled (*n* = *16* with a benign nodule, *n* = *17* with a malignant nodule). Following isolation and amplification of plasma cfDNA and matched peripheral blood mononuclear cells (PBMC) from patient blood samples, WES of cfDNA and PBMC DNA was performed. After genomic alignment and filtering, we looked for lung-cancer associated driver mutations and next identified high-confidence somatic variants in both groups.

**Results:** Somatic cfDNA mutations were observed in both groups, with the cancer group demonstrating more variants than the benign group (1083 ± 476 versus 553 ± 519, *p* < 0.0046). By selecting variants present in >2 cancer patients and not the benign group, we accurately identified 82% (14/17) of cancer patients.

**Conclusions:** This study suggests a potential role for cfDNA for the early identification of lung cancer in patients with CT-detected pulmonary lesions. Importantly, a substantial number of somatic variants in healthy patients with benign pulmonary nodules were also found. Such “benign” variants, while largely unexplored to date, have widespread relevance to all liquid biopsies if cfDNA is to be used accurately for cancer detection.

## Introduction

Lung cancer is the leading cause of cancer-related death in the United States (1). Most patients present with advanced disease with limited cure potential. The National Lung Screening Trial (NLST) demonstrated a 20% relative reduction in cancer mortality with lung cancer screening with low-dose computerized tomography (LDCT) compared to radiography (1). However, CT poses limitations; namely, the high incidence of indeterminate pulmonary nodules (IPNs). Over 24% of NLST participants in the CT arm had IPNs, and the majority (>96%) were false positive (1). IPNs are also common in routine clinical radiology, with frequency up to 60%. Patients with IPNs often require long-term sequential imaging and/or biopsy to establish diagnosis (2).

There has been growing interest in plasma cell free DNA (cfDNA), or “liquid biopsy,” for cancer diagnosis and surveillance (2, 3). cfDNA is thought to be released into circulation by necrotic or apotpic cells, and is frequently present at higher quantities in patients with cancer than healthy individuals (4–6). For example, Sozzi et al. demonstrated 20-fold higher levels of cfDNA in non-small cell lung cancer (NSCLC) patients than control patients (7). However, cfDNA quantity alone is not a sufficient diagnostic biomarker because of variability and overlap in concentrations between patients with and without cancer (4, 6, 8, 9). As such, mutational analysis of cfDNA to identify tumor variants may be more suitable for cancer diagnosis and monitoring (10, 11).

To date, studies have not evaluated cfDNA for the characterization of CT-detected IPLs as malignant or benign for early lung cancer detection. Current cfDNA studies for lung cancer largely explore driver mutations from the primary tumor and do not address the broader genomic landscape achieved by next generation sequencing (NGS)-based whole genome and/or exome sequencing (WGS/WES) (3, 11–13).

Our purpose in this pilot investigation was to perform WES of plasma cfDNA and matched peripheral blood mononuclear cell (PBMC) germline DNA in patients with a CT-detected pulmonary nodule to determine if somatic cfDNA mutations can differentiate malignant from benign pulmonary nodules.

## Methods

### Study Population

This HIPAA-compliant retrospective study was approved by the Duke University Health System's Institutional Review Board (IRB). 33 patients were randomly selected from our laboratory's repository that enrolls patients with a CT-detected IPL, seen in our surgery, oncology, pulmonary, and medicine clinics. All patients enrolled in this repository provide written informed consent, and following consent, patients provide blood specimens for plasma/PBMC isolation.

For this pilot study, we randomly selected 17 patients with a proven malignant pulmonary lesion, and 16 patients with a proven benign pulmonary lesion. Inclusion criteria included: ≥40 years of age; no prior history of malignancy; chest CT(s) available for radiologist review; baseline CT with ≥4 mm IPL (NLST size criteria); and blood specimen collection within 100 days of the baseline CT. Patients in the cancer group had a pathologically proven primary lung malignancy. Control patients had a benign nodule determined by: pathology (wedge/needle biopsy); or follow-up CTs demonstrating nodule resolution; or ≥2 years stability by sequential CT imaging and clinical data.

### CT Review

A board-certified thoracic radiologist reviewed CT lesion features: size, consistency (solid, part-solid, or ground glass), margins, and cavitation. For control (benign) patients not receiving biopsy, all sequential CTs were reviewed to assess lesion resolution and/or stability for ≥2 years.

### Plasma Sampling

Blood specimens were collected in K_2_EDTA Vacutainer tubes (purple top) for plasma isolation and lithium heparin tubes (green top) for PBMC isolation. For plasma isolation, tubes were centrifuged at 820 x g for 10 min at 4°C, and the supernatant fraction transferred to a fresh tube and re-centrifuged at 10,000 x g. The supernatant fraction from the second centrifugation was transferred to cryotubes for storage in a −80° C freezer in our laboratory. PBMCs were isolated using Ficoll-Paque Plus (GE Healthcare 17-1440-02) per the manufacturer's instructions. PBMCs were counted, pelleted by centrifugation, and pellets resuspended in Bambanker freezing medium (LYMPHOTEC, Inc., Tokyo, Japan) for storage at −80°C.

### DNA Extraction, Amplification, and Sequencing

Prior to study initiation, a standard operating procedure (SOP) for DNA extraction, amplification, and sequencing was established. Briefly, plasma cfDNA was isolated from plasma using the QIAamp circulating nucleic acid kit (Qiagen). Germline DNA was isolated from PBMCs using the QIAamp DSP DNA Mini Kit, version 2 (Qiagen). Sufficient DNA was obtained from all samples (minimum requirement for further manipulation is 10 ng DNA at concentration of >200 ng/μl). DNA was amplified using the REPLI-g kit (Qiagen). Sequencing libraries were prepared for 66 samples (33 plasma cfDNA, 33 matched PBMC DNA) using the Agilent SureSelect All Exon V5+UTR kit for the HiSeq2500 (Agilent Technologies, Inc., Santa Clara, CA). The target size of this kit is 75 Mb and covers 359,555 exons in 21,522 genes. Amplified DNA (3 μg of each sample) was fragmented using the Covaris S-series single tube sample preparation system (Covaris, Inc. Woburn, MA). A quality check before and after fragmentation was performed on the AATI Fragment Analyzer (Advanced Analytical Technologies, Inc., Ames, Iowa) using a Standard Sensitivity Genomic DNA Analysis kit (LabGene Scientific), according to manufacturers' protocols. DNA was fragmented to a desired size range of ~150–220 bp. Library preparation was carried out according to the SureSelect^XT^ Target Enrichment System for Illumina Paired-End Sequencing Library Protocol (version 1.5, November 2012) (Agilent). After adapter ligation, DNA was hybridized to biotinylated bait oligonucleotides homologous to the exon regions of the genome. After hybrid capture, target DNA was amplified with addition of index tags, prior to pooling and generation of 125 bp paired end sequence on the HiSeq2500 (Illumina, Inc., San Diego, CA), using version 3 chemistry. Exome sequencing (plasma cfDNA and PBMC DNA) was obtained for all patients to mean depth 49X (minimum 40X) with 82% coverage of the exome.

### DNA Analyses

The quality of sequence reads was evaluated using FastQC (14), including the aspects of per base quality, GC content, Kmer content, sequence length distribution, sequence duplication levels, and overrepresented sequences. The paired-end reads were aligned to the human genome, b37 version with decoy sequences, to improve mapping and variant discovery. Mapping quality was assessed using SAMtools flagstat (15). We applied Genome Analysis Toolkit (GATK) pre-processing steps to the bam files (16). First, duplicated reads were marked and removed using the Picard tool (17). Then, local realignment around indels was performed to correct mapping-related artifacts. Base quality scores were recalibrated to correct sequencing errors and other experimental artifacts. The target sequencing regions with 100 bp flanking sequences added were supplied to speed up the process and reduce false positive calls. An additional realignment step was performed using the plasma DNA sample and its matched PBMC DNA sample together to ensure consistent alignment for the two DNA samples from the same patient.

Using SAMtools mpileup, an mpileup file was generated for each patient's matched pair of plasma/PBMC DNA. Alignments with mapping quality <20 were skipped. Mutations (single nucleotide variants (SNVs) and indels) were called using VarScan2 in “somatic” mode (18). We were only interested in the positions where the base of the plasma cfDNA did not match that of the PBMC DNA. Variants with >90% strand bias were removed. A subset of high-confidence variants was selected, with an allele frequency >1·5% for plasma DNA, allele frequency ≤1% for PBMC DNA, and Fisher's exact test *p*-value <3%. An additional filter was applied to select variants having at least 3 reads containing the variant allele, a minimal read depth of 20, a minimal variant allele frequency of 1.5%, and a minimal average base quality score of 30. False positives due to systematic artifacts were removed using bam-readcount and the FPfilter accessory script.

Final variants were annotated using ANNOVAR with build hg19 databases (19), including refGene, dbNSFP version 2.6, COSMIC database version 70, NHLBI-ESP project with 6500 exomes, 1000 Genomes Project, dbSNP 138, CLINVAR database with variant clinical significance and variant disease name, etc. The annotated variants were filtered to remove those found in the dbSNP 138 database, except for those also observed in the COSMIC database, and to keep non-synonymous exonic or splicing events. Further filtering was performed by retrieving the flanking sequences around the mutation sites, and removed the indels called in or close to homopolymeric regions, which are likely false positives due to Illumina sequencing artifacts. We also collected all of germline mutations called by VarScan2, and removed those leftover germline mutations from our mutation list.

Downstream analyses first consisted of looking for multiple driver mutations commonly found in the exomes of lung cancer patients: AKT1, ALK, BRAF, DDR2, EGFR, ERBB2, FGFR1, FGFR3, KRAS, MAP2K1, MET, NRAS, NTRK1, PIK3CA, PTEN, RET, RICTOR, and ROS1 (20). Second, we calculated the total number of mutations for each patient and compared the total number of these variants, as well as the number of mutations of each function type, between groups.

### Statistical Analyses

A two-sample *t*-test was used to compare the mutation quantities between groups. For each variant, we calculated the number of patients in each group having that variant or not having that variant. We then selected variants observed in ≥2 patients from the malignant group, but not in the benign group. After checking the bam files in IGV, we removed false positives and kept the final 10 mutations and plotted them in a heatmap with two-way hierarchical clustering using the Jaccard distance measure and the Ward's linkage method. For CT features, a two-sample-test was used to compare nodule size between groups. The other CT features were compared between groups with Fisher's exact test.

## Results

### Study Demographics

Demographics are summarized in Table 1. Thirty-three patients were enrolled (*n* = *17* cancer; *n* = 16 benign). There was no significant difference in pack-years smoked between groups (*p* = 0.201). All patients in the cancer group had NSCLC.

**Table 1:** Patient demographics and clinical profiles.

	**Group**	
**Demographic**	**Cancer** (***n*** **=** **17)**	**Control** (***n*** **=** **16)**
Age, years	67.29 ± 6.9	64.5 ± 10.7
Range	55–80	47–84
Gender		
Male	5	8
Female	12	8
Smoking pack years	37.4 ± 30.0	23.5 ± 21.3
Stage		
I	8	
II	2	
III	5	
IV	2	
Histology		
Adenocarcinoma	10	
Squamous cell carcinoma	6	
Large-cell neuroendocrine carcinoma	1	

### CT Features

Table 2 summarizes CT features of patient's pulmonary lesions. Size was the only CT feature that differed between both groups, with the cancer group exhibiting larger diameter than the control group (average 3.8 ± 2.7 cm vs. 1.4 cm ± 1.0, *p* < 0.002).

**Table 2:** CT imaging characteristics of pulmonary lesions.

	**Cancer (*n* = 17)**	**Control (*n* = 16)**	***P*-value**
Lesion size (cm)	3.8 ± 2.7	1.4 ± 1.0	0.002^*^
Cavitation	5 (29.4%)	2 (12.5%)	0.398
Spiculated margin	7 (41.2%)	2 (12.5%)	0.118
Subsolid composition	1 (5.9%)	2 (12.5%)	0.601

* *indicates statistically significant (p < 0.05)*.

### cfDNA Analysis

**Figure 1:**
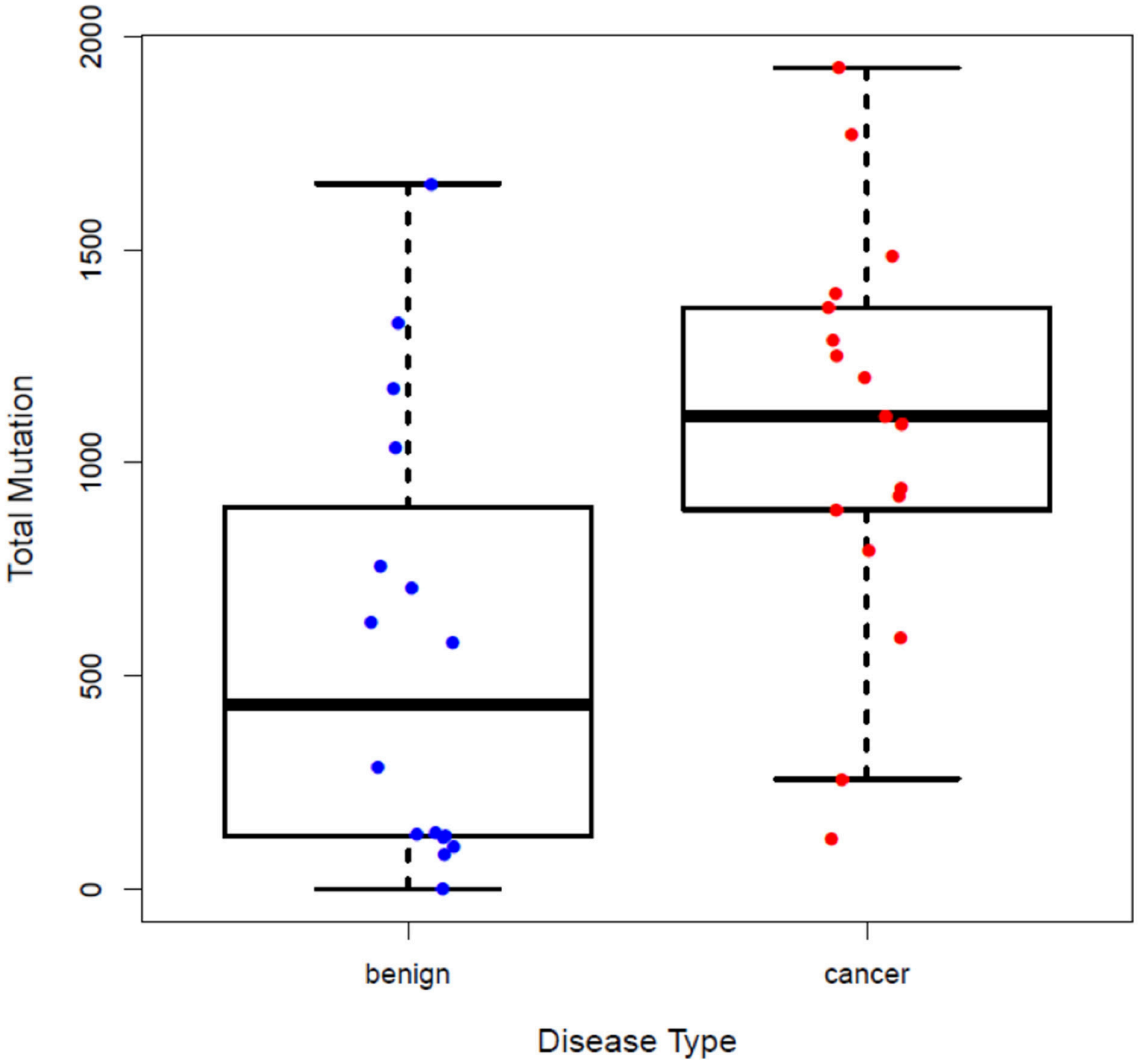
Number of somatic variants detected in the plasma cfDNA of patients in the control and cancer groups. More somatic cfDNA variants were observed in the cancer group than the control group, *P* = 0.0046.

**Figure 2:**
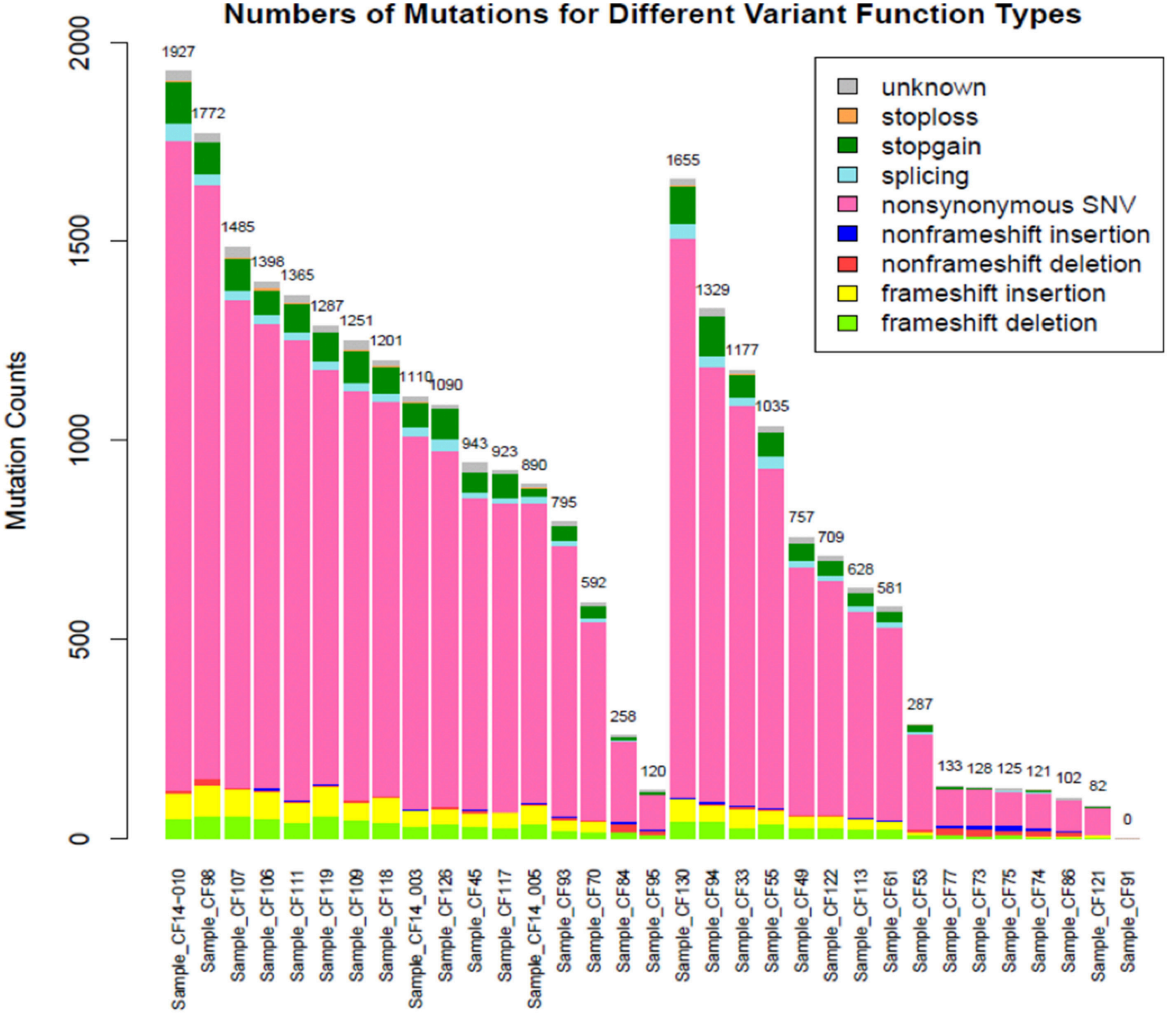
Number of mutations for different functional types for the cancer **(left)** and control **(right)** groups.

Subjects' blood specimens were collected for plasma cfDNA and PBMC germline DNA isolation within a mean of 15.4 days (standard deviation, 26.0 days) of the baseline chest CT examination. Following variant calling, annotation, and filtration, high-confidence somatic cfDNA variants (those observed in plasma cfDNA, but not in matched PBMC germline control) were identified. The number of variants was higher in the cancer group than the control group (1083 ± 476 vs. 553 ± 519, *p* < 0.0046) (Figure 1). We summarized the number of somatic cfDNA variants for each function type per patient in both groups (Figure 2). Non-synonymous SNVs were observed with highest frequency in each group.

**Figure 3:**
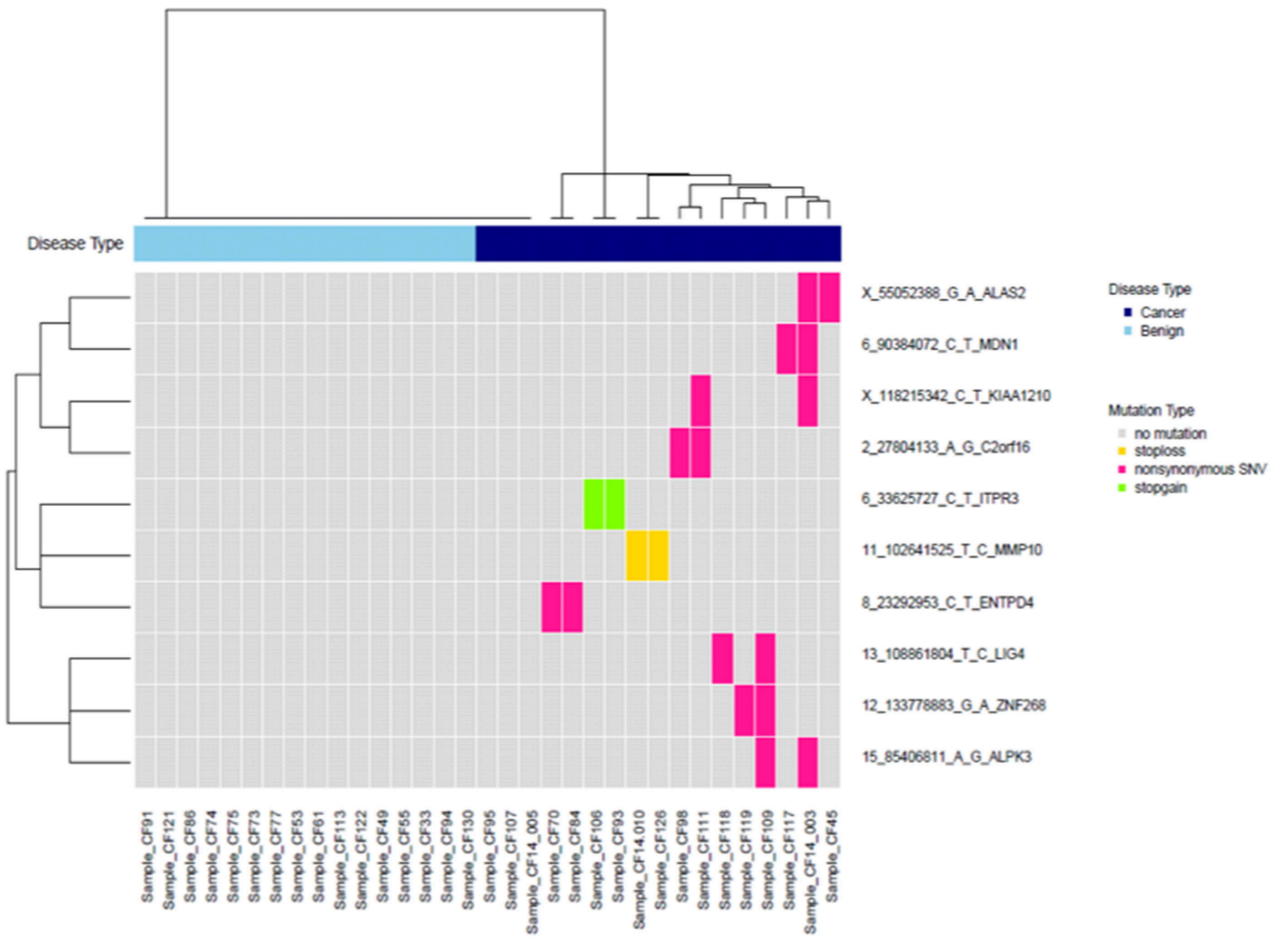
Clustering of 10 high-confidence somatic variants observed in at least two patients with cancer, but not in the control group.

We selected variants that were observed in ≥2 cancer patients but not in control patients. After checking the bam files in IGV and removing false positives, this yielded ten variants, which are plotted in a heat map with two-way hierarchical clustering in Figure 3. With these ten variants, we identified 82% (14/17) of cancer patients.

## Discussion

CT detected pulmonary lesions pose a significant challenge in chest medicine (21). While certain CT features, such as spiculated margins and cavitation, are more common with malignant lesions, these features have limited diagnostic accuracy (22). This dilemma is herein underscored, where lesion size was the only CT feature differing between groups (Table 2). Owing partly to the potential for early cancer detection, there is growing interest in peripheral blood biomarkers, including cfDNA, for cancer diagnosis and monitoring (5, 7, 11, 23). NGS of cfDNA has many potential benefits, including alleviating need for biopsy, definition of a tumor's genomic signature at the nucleotide/indel level to identify variants beyond pre-defined targets, and analysis of a tumor's sub-clonal population/heterogeneity (10, 12, 13, 23, 24). However, standardized methods for NGS/WES and cfDNA analysis for clinical diagnostics are lacking. Additionally, the extent to which plasma cfDNA mutations are consistently found in patients with primary lung cancer has not been systematically investigated.

We performed WES of plasma cfDNA and individually-matched PBMC germline DNA in 33 patients with IPNs, who were followed to diagnosis of lung cancer (*n* = 17) or benign nodule (*n* = *16*). We achieved several objectives: (i) identification of somatic cfDNA mutations in patients with malignant lesions; (ii) identification of many somatic mutations in patients with benign pulmonary lesions; (iii) and preliminary demonstration of the ability of cfDNA to differentiate malignant from benign pulmonary lesions. Additionally, and perhaps more importantly, our study underscores key challenges and pitfalls for plasma cfDNA sequencing for early cancer detection before specific somatic mutations from the primary tumor are known. This is a different clinical scenario than prior studies, which report the utility of plasma cfDNA for monitoring response to therapy when specific mutations are first identified in the primary tumor.

While there were more somatic cfDNA variants in patients harboring malignant pulmonary nodules than those with benign nodules, the number of variants observed in the benign group, most of which were non-synonymous SNVs, was not negligible. At present, the mutational landscape of normal human tissues, and their age-related variation, is largely unknown. A growing body of literature suggests a spectrum of somatic aberrations in normal and/or non-cancerous tissues (25–27). Using massively parallel WGS, Hoang, et al. demonstrated substantial somatic genetic variation in normal tissues that increases with age, and varies with tissue type, DNA repair capacity, and environmental exposure (24, 25, 28). In another study, ~11% of 123 non-cancer patients carried *TP53* mutations (28). Other data suggests that at least half of the somatic variations in tumors originate before tumor initiation; such “passenger” variants may be sequela of normal aging/development, confer no clonal advantage, and lack a direct relationship with tumorogenesis (26). Herein lies a major challenge of genomics for cancer screening or detection—until more is known about the genetic landscape of normal tissues, the temporal progression of normal tissues relating to age, self-renewal, and successive DNA repair, and the degree to which seemingly “cancer-specific” mutations overlap with normal tissues, it is difficult and problematic to establish confident diagnostic accuracy.

WGS/WES of cfDNA theoretically detects genetic variation at a nucleotide level and allows analyses of subclonal tumor populations (26). However, it is doubtful that all plasma cfDNA mutations arise from tumors. Dietz et al. demonstrated that WES of cfDNA and matched tumor samples in NSCLC patients revealed only modest and variable concordance between somatic variants in cfDNA and tumor samples (~5–57%, median 17·2%) (12). Further, cfDNA revealed mutations not present in synchronous tumors, including an *MTOR* mutation in one patient with allele frequency 15% (12). While it is possible that such cfDNA variants derived from non-sampled tumor populations or distant metastases, the plausibility that such aberrations originate from normal cells, unrelated to the tumor, remains. While comparison with individually-matched germline DNA and high fidelity filtering using databases like dbSNP, as performed here, theoretically removes germline variability, until more is known about genetic aberrations in normal tissues and their expression in cfDNA, the specificity of cfDNA genomics remains challenged (3).

NGS offers the potential for broad sequencing coverage, which may enumerate tumor heterogeneity, a hallmark of nearly all (if not all) tumors (26). However, in addition to coverage breadth, the ideal method must be sensitive enough to detect low frequency mutations (24). This is critical if cfDNA is to be used advantageously for early cancer diagnosis or for prompt detection of treatment-altering therapy resistant sub-clones (2, 23). Amongst other factors, allele frequency, sequencing read depth, and sequencing errors limit the sensitivity of variant detection. Mutant allele frequencies in cfDNA may be as low as 0.01% (23). While refined digital PCR (dPCR) and multiplexed assays for single locus examination have reported sensitivities as low as 0.001%, most off-the-shelf multiplexed panels for targeted sequencing carry an ~1% limit of detection (2, 13, 23, 26). Although higher resolution (<0.01%) is reported with customized amplicon-based panels, the ability to detect low frequency variation amongst a milieu of “normal” or non-tumorogenic variation is a limitation of cfDNA with relevance to clinical translation for cancer diagnosis and management (2).

While we correctly identified 82% (14/17) of the cancer patients using ten high-confidence somatic cfDNA variants, our study was limited by read depth. The achieved depth (mean 49X) was too low to capture all variation in the tumor's exome. This may partly explain why driver mutations, including EGFR, ALK, and KRAS, were not identified in lung cancer patients. Other contributory explanations causing potential false negatives in this regard include the small study size and/or low prevalence of driver mutations in the tumor or cfDNA. Additionally, because of the small sample size, the variation of somatic mutations with tumor stage could not be investigated. Nonetheless, the potential for deep sequencing of cfDNA to identify rare somatic mutations unique to malignant pulmonary nodules supports its value as a potential biomarker for early lung cancer detection.

As with all NGS platforms, artifacts introduced during sequencing may contribute to false positives and limit diagnostic accuracy. PCR amplification is ubiquitous to nearly all high-throughput sequencing methods, particularly for cfDNA, where genome equivalents or allele frequency may be low. However, amplification introduces point mutations; and while true DNA mutations should be present on both strands of the original DNA duplex, it is virtually impossible to distinguish mutations deriving from the original DNA from point errors covering the same locus introduced during library amplification (2, 25). Additionally, the uniformity of amplification (i.e., degree to which the library is amplified equally) is variable (29). In this regard, molecular tags or barcodes, which tag fragments deriving from the initial PCR cycle, improve sensitivity and accuracy of consensus sequencing (30, 31). While barcoding has conventionally been used for targeted loci, recently a bottleneck sequencing system, using a molecularly barcoded library for high sensitivity (<10^−9^ per base pair mutation detection) and unbiased WGS was described (25). Such a system would improve sensitivity and specificity of WES/WGS, and potentially exploit the full potential of NGS for cancer detection and monitoring. As such, future work will combine deep sequencing (>1000x) with a barcoded library to detect high-confidence variants for early lung cancer diagnosis.

In summary, our results suggest a potential role for WES of cfDNA for early lung cancer detection in patients with CT-identified pulmonary lesions. While this is a small retrospective study and the widespread implications of individual somatic mutations identified herein remain largely unknown, this investigation elucidates key considerations and challenges affecting diagnostic accuracy of cfDNA analyses. Further, this study demonstrates a non-negligible number of somatic mutations in patients with benign lung nodules. The latter, an area largely unexplored to date, has widespread relevance if genomic biomarkers are to complement imaging in early lung cancer detection.

## Ethics Statement

This retrospective study was approved by the Duke Institutional Review Board (IRB). We utilized blood samples from the senior investigator's (Dr. Patz) laboratory repository, and all patients who participate in this research repository consent to this blood sampling.

## Author Contributions

TDT and EFP contributed to research plan and synthesis, study design, imaging review, manuscript preparation. MC contributed to data analysis and collection, sample preparation, and manuscript preparation. SG contributed to manuscript review. JW and XR contributed to data and statistical analysis and manuscript review.

### Conflict of Interest Statement

The authors declare that the research was conducted in the absence of any commercial or financial relationships that could be construed as a potential conflict of interest.
